# An evolutionary behaviorist perspective on orgasm

**DOI:** 10.3402/snp.v6.32130

**Published:** 2016-10-25

**Authors:** Diana S. Fleischman

**Affiliations:** Department of Psychology, University of Portsmouth, Hampshire, United Kingdom

**Keywords:** orgasm, evolutionary psychology, behaviorism, shaping, reinforcement, punishment, classical conditioning, operant conditioning, signaling

## Abstract

Evolutionary explanations for sexual behavior and orgasm most often posit facilitating reproduction as the primary function (i.e. greater rate of fertilization). Other reproductive benefits of sexual pleasure and orgasm such as improved bonding of parents have also been discussed but not thoroughly. Although sex is known to be highly reinforcing, behaviorist principles are rarely invoked alongside evolutionary psychology in order to account for human sexual and social behavior. In this paper, I will argue that intense sexual pleasure, especially orgasm, can be understood as a primary reinforcer shaped by evolution to reinforce behavior that facilitates reproductive success (i.e. conception through copulation). Next, I will describe an evolutionary account of social shaping. In particular, I will focus on how humans evolved to use orgasm and sexual arousal to shape the social behavior and emotional states of others through both classical and operant conditioning and through both reproductive and non-reproductive forms of sexual behavior. Finally, I will describe how orgasm is a signal of sensitivity to reinforcement that is itself reinforcing.

Imagine the pleasure of seeing a cute, happy baby; sitting in the warm sunshine; eating something fatty and sugary; or getting relieved of a few minutes of low-voltage intermittent shocks. You would likely estimate the pleasure of orgasm as greater than any of these things. Evolution has used orgasm to train us toward adaptive behavioral ends; orgasm and high sexual arousal are currencies that tap directly into bliss states. Reinforcement and reward are better motivators of behavior and are better at shaping new behaviors than punishment (Pryor, 1999; Skinner, 1938). Given these two principles, the thesis in this paper is that (1) evolution has used orgasm to promote adaptive behavior including non-reproductive sexual behavior, (2) we have evolved to use orgasm and sexual arousal to shape one another's behavior, and (3) orgasm serves as a signal to another person of devotion, vulnerability, and malleability, which is, in itself, reinforcing. This paper won't go into how orgasmic pleasure works or the evidence that orgasm facilitates reproduction directly (see Wheatley and Puts (2015) for a good overview and other papers in this volume). Instead, I will consider orgasm the pinnacle of a continuum of highly reinforcing sexual pleasure.

## Orgasm and sexual behavior as primary reinforcers

Animals have different primary reinforcers depending on which cues are most likely to be statistically associated with adaptive outcomes. A reinforcer is any stimulus that increases the frequency, duration, or intensity of a behavior (Schultz, 2015). Organisms that evolve to experience pleasure as a result of engaging in adaptive behavior will be more motivated to engage in such behavior. When a reward or ‘primary reinforcer’ is paired with a given stimulus, that stimulus becomes a secondary reinforcer. For example, when a dog is clicker trained, he hears a click when he is given food for a given behavior. The click becomes a secondary reinforcer that can motivate future behavior because it has been paired with food, a primary reinforcer (even after food is no longer paired with the click). Orgasm, in behaviorist terms, is a primary reinforcer, a pleasurable unconditioned stimulus that, without any classically conditioned association, is inherently and innately reinforcing. Orgasm, like other primary reinforcers, can make stimuli that are paired with it (e.g. a color, someone's face, a specific smell) reinforcing. Food, warmth, sex, and sleep are examples of primary reinforcers and these are evolutionarily conserved among many organisms. Because evolved psychological mechanisms solve adaptive problems with biologically prepared inputs and because so many cues have been recurrently associated with these inputs, there are likely many primary reinforcers that exist for humans that don't exist for animals.

Consider evolution as an agent that calibrates the subjective pleasure of behavior based on how adaptive it is for the organism. It's no wonder that finding and consuming food and sex are primary reinforcers as they facilitate survival and reproduction. However, considering sex as reinforcing can be taken one step further. According to an adaptationist account of conditioning, any cues of courtship or reproduction that are consistently associated with higher reproductive success recurrently throughout evolutionary history can become a primary reinforcer. This can include practicing courtship skills (e.g. telling stories), the attention one gets from potential mates (e.g. eye contact, smiling), initiating sexual behavior (e.g. intimate touch, kissing) all the way up to penetration, orgasm, and seeing one's partner indicating intense pleasure and orgasm.

Another prediction derived from an evolutionary perspective is that primary reinforcers differ over the lifespan and that different primary reinforcers have different salience depending on the adaptive problems that were recurrently faced by our ancestors during particular life stages. For instance, parental praise will be a primary reinforcer for children but less so for adults. Attention (e.g. eye contact, orienting body language) from attractive members of the opposite sex should be more of a primary reinforcer for reproductive-aged men or women than children. We should also expect this with sex; sexual behavior will yield the most pleasure when it is fulfilling adaptive goals such as improved bonding between parents or forming a new bond with someone of high status. For example, behaviorists would argue that a smile is a secondary reinforcer because it has been classically conditioned by being paired with food or another primary reinforcer. An evolutionary learning perspective might differ. Experimental evidence shows that infants, 2–7 months old, can be conditioned to prefer sounds using a smile as reinforcement (Routh, 1969). This indicates a smile may be an innate positive reinforcer or, at least, that infants are biologically prepared to associate a smile with primary reinforcers like food or warmth. For infants, the major adaptive problem they face is having an engaged and motivated caretaker who will provision them and protect them from harm; a smile is a cue that this adaptive problem is being solved by the behavior of the infant.

More salient to the topic of orgasm, pictures of nude adult women would be a strong primary reinforcer for men after puberty but would probably not be a primary reinforcer for prepubescent males. Researchers have found that male rhesus macaques will ‘pay’ (give up part of a juice reward) to view the genitals of female macaques or the faces of high status males but will not ‘pay’ to view the faces of low status conspecifics (Deaner, Khera, & Platt, 2005). Another study found that peak fertility female macaques preferred to view the faces of male conspecifics (Lacreuse, Martin-Malivel, Lange, & Herndon, 2007) and showed reduced preference during periods of lower fertility. These studies show that social information in and of itself can act as a primary reinforcer. For the sexually inexperienced male rhesus macaques, both the photos of the high status male's faces and the genitals of the females had not previously been associated with food, sex, or other positive reinforcement (i.e. not classically conditioned) (Michael Platt, primatologist, April 7, 2016 personal communication). An evolutionary perspective would predict that sexual primary reinforcers do not show equipotentiality; they are differentially rewarding depending on how adaptive they are to an organism. This may be contingent on age, sex, and fertility status. While stimuli like nudity may or may not be innate or a primary reinforcer, we should expect that for stimuli that are very salient to reproduction, there is at least a biological preparedness for associating sexual stimuli with pleasure, thereby increasing their reinforcing qualities. The sex of the organism should also be an important variable influencing the valence of sexual pleasure.

## An adaptationist account of sex differences in sexual reinforcement

Sex differences in orgasmic capacity and sexual pleasure can be explored within an evolutionary behaviorist framework. We infer from the presence of ejaculation and context-dependent facial expressions that in many other species, only males consistently experience orgasm during sexual behavior (Ferro, 2013; Lloyd, 2009). Ejaculation could be associated with no more pleasure than urinating or defecating if ejaculation was not necessary for reproduction. An adaptationist perspective explains why males consistently experience the peak pleasure of orgasm from intercourse more than other sexual activities; orgasm motivates men toward the most adaptively important outcomes. However, men also consistently reach orgasm more easily when engaging in other forms of sexual behavior. Men's gametes (sperm) are much less costly to produce and men have much lower obligate parental investment than women (Trivers, 1996). For men to be reproductively successful, they need, on the low end, to only engage their time and resources as much as is necessary to have sex with a woman.

Consider men's rate of orgasm in light of Error Management Theory (Haselton & Buss, 2000) also known as the Smoke Detector Principle (Nesse, 2001). If there are two kinds of errors an organism can make, evolution will bias behavior toward the less costly of these errors. If you design a smoke alarm, it's better for it to be calibrated to go off in error when there is no fire than it is for it to remain silent when there is a fire. For men, sexual behavior has low potential costs compared with great potential reproductive benefits. This is why men are calibrated to over-perceive sexual interest on the part of women (Perilloux, Easton, & Buss, 2012). For the same reason, we should expect men to experience orgasm in a variety of conditions, even if they do not lead directly to fertilization. Experiencing orgasm when engaging in an act that cannot result in reproduction is a less costly error than not experiencing orgasm during an act that can lead to conception (Figueredo et al., 2005).

There is evidence for this both in humans and animals. Semen can be collected easily in many birds and mammals, by providing a male with a crude facsimile of a female or vagina (Rouge & Bowen, 2002). Men, more than women, are prone to fetishes and paraphilias, and easily pair sexual arousal with a given stimulus through classical conditioning (Rachman, 1966). However, just because men are more likely to reach orgasm with myriad forms of sexual behavior and with different sexual partners does not mean that orgasm or sexual pleasure will be equal across these contexts. We should expect more adaptive conditions to lead to greater sexual pleasure and more intense orgasm (more on this later). However, we should also expect plasticity built into the sexual motivation system through reinforcement. Men have preferences for young, fertile women and various forms of attractiveness that signal health and reproductive value (Sugiyama, 2005). However, men will experience orgasm and sexual pleasure with women that are available to them even if they do not evince these cues. In terms of fertilization, evolution should maximize orgasmic pleasure for men who are with partners most likely to conceive, that is, women of reproductive age who are healthy enough to carry a pregnancy, but men will also be motivated with a history of orgasmic reinforcement toward women who will be more likely to choose them repeatedly as mates.

Many scientists have been puzzled by women's orgasmic frequency. Why don't women consistently experience orgasm and, conversely, why do women have orgasms at all? The fact that women experience orgasm, at least some of the time, is one piece of evidence that orgasm is a primary reinforcer for some kinds of adaptive behavior. Some (e.g. Prause, 2011) have speculated that it is precisely because orgasm is variable in women that it may be more reinforcing than it is for men; variable reinforcement has been found to be a greater driver of behavior that is resistant to extinction than consistent reinforcement (e.g. gambling on a slot machine is more resistant to extinction than pulling a lever with a consistent payout) (Pryor, 1999). For women, sex is much more costly both in terms of potential parental investment and sexually transmitted disease risk and thus we should expect evolution to be more selective about the sort of sexual behavior that should be reinforced with extreme pleasure.

In order for a woman to be reproductively successful, she must carry a child for 9 months and, until recent history, breastfeed for another 3 years. Women can have far fewer offspring than men over their lifetimes, have less variable rates of reproduction, and have little reproductive incentive to have sex with more men. A woman who has intercourse with 100 men in a year will, on average, have no more offspring than a woman who has regular sex with one fertile man. Moreover, women are much more susceptible to sexually transmitted infections than men and have much greater disease burden (e.g. sterility) as a result of these infections (Madkan, Giancola, Sra, & Tyring, 2006). For these reasons, copulation isn't always adaptive and a woman who was easily orgasmic in a variety of conditions could be making a more costly error: being motivated to engage in behavior that is unlikely to result in the optimal reproductive outcome of conceiving with a genetically fit male who is free of disease (Miller, 2000).

Thus, it is useful to consider what kinds of sexual activity evolution would want to orgasmically reinforce in women for fertilization and conception versus for bonding. I speculate that this disease risk and the concomitant focus of sexual pleasure away from the vaginal mucous membranes (which are more likely to transmit disease upon contact than the clitoris and external vulva) might have been a driver toward the variety of sexual practices (e.g. frottage) humans engage in that do not involve copulation. Further evidence for this is that most women cannot achieve orgasm through vaginal intercourse alone (Lehmiller, 2013; Lloyd, 2009) and most women do not consider the vagina to be the most important erogenous zone compared with the clitoris (S. Fisher, 1973). Later, we will explore how orgasm may be adaptively calibrated for bonding between parents and social benefits that translate into increased reproductive success (e.g. formation of alliances that increase status).

## Sexual pleasure calibrated to maximize fertilization

Pleasure and primary reinforcement are not binary phenomena. We should expect pleasure to be gradient and calibrated to the scarcity or adaptive value of the stimulus. Drinking water is more pleasurable when one is thirsty and eating high-calorie foods – especially those that have nutrients that were scarce throughout evolutionary history (e.g. salt, sugar, fat) – feels more pleasurable than eating foods that were abundant or offer less nutritional value. Orgasm may be similarly calibrated and sensitive to contexts of scarcity or abundance. For both men and women, we should expect sex and orgasm to feel more pleasurable when mates or the opportunity for sexual contact are scarce. We should predict that sexual contact with healthy and attractive conspecifics to yield more pleasure. Aspects of health and attractiveness are normally distributed. Thus, we can expect that health and attractiveness increase the pleasure and reinforcing quality of sexual behavior not only because they are statistically associated with greater fertility and therefore greater reproductive success but also because those sexual partners are necessarily more scarce than partners more average on genetically endowed qualities. Evolution has used orgasm to reinforce behaviors that are directly related to fertilization such as an orgasm associated with ejaculation during intercourse. However, it's also clear that orgasm facilitates the motivation to engage in other non-reproductive sexual behaviors such as oral sex, masturbation, and same-sex sexual behavior.

Let us first consider how orgasm could be calibrated to help women gain genetic benefits that would make each of their costly offspring more likely to be healthy and reproductively successful. There is controversial evidence (Wheatley & Puts, 2015) that female orgasm facilitates conception (e.g. by dipping the cervix into the seminal pool); however, orgasm need not facilitate conception directly for the purposes of increasing reproduction with certain males but instead could reinforce repeated sexual behavior with those males. How would evolution optimally calibrate female orgasm to motivate women to have conceptive sex? Some evidence has shown that women are more likely to have orgasms with men who show good genetic quality. Attractiveness, masculinity, and symmetry have been associated with health as they are expensive signals to produce (Rantala et al., 2012; Rhodes Chan, Zebrowitz, & Simmons, 2003; Zahavi, 1975); Some studies have shown that women are more likely to have orgasms with men who are masculine and symmetrical (Puts, Welling, Burriss, & Dawood, 2012; Thornhill, Gangestad, & Comer, 1995). The vigor, health, coordination, and theory of mind that aid in facilitating orgasm in a partner are all costly signals of health and fitness as well (Miller, 2000).

The ‘sexy sons’ hypothesis (R. A. Fisher, 1930) posits that females should choose males who are likely to produce male offspring that other females will find sexually attractive. Traits that are attractive to females are often but not necessarily associated with health. For instance, women who prefer longer penises are more likely to have vaginal orgasms (Costa, Miller, & Brody, 2012) demonstrating a drive to mate with males who may produce sons that other females prefer but not with a trait that has (thus far) been shown to correlate with health or quality (But see also evidence that erectile function correlates with health, e.g. Miller, 2015). Thus, orgasm may serve to reinforce engaging in repeated sex with men who are likely to produce such sexy sons, regardless of whether these traits signal health or quality.

## Orgasm as reinforcement to facilitate pair bonding and social bonds

Thus far, we have mostly considered the idea that orgasm reinforces sexual behavior toward conception. This may have been the reason why orgasm initially evolved to be pleasurable and seems to be the motivating force for most males of other sexually reproducing species. It has long been theorized that the aseasonal (i.e. no distinct period or season of fertility and no estrus) and high sex drive of humans (i.e. not strongly tethered to fertility) is indicative of its evolution as a mechanism for reinforcing bonds between two people. These bonds have been discussed especially with regard to their importance between parents provisioning for offspring (Marlowe, 2000). Although this discussion will focus on parental bonding, ‘pair bond’ in this paper can indicate any attachment between two people whether monogamous or polygynous, reproductive or non-reproductive, heterosexual or homosexual.

If evolution designed a woman to have sex with a man for his superior genetic complement or a man to have sex in order to conceive offspring, they need not be motivated to engage in sex longer than is needed for fertilization and certainly would not be motivated to engage in non-reproductive sex. Indeed, for many researchers, bonding is seen as the primary function of female orgasm; ‘orgasm serves as a secondary reinforcer linking sexual behaviors and partner affiliation’ (Prause, 2011). However, others see sire choice as its primary mechanism (Puts, Dawood, & Welling, 2012). In practice, because the characteristics of good genes, good mates, and good social partners often overlap (e.g. strength, empathy, health), it is often difficult to disentangle these influences.

If orgasm functions primarily to form and maintain pair bonds, especially between parents, this leads us to expect somewhat different design features of orgasm than those for the benefit of merely facilitating reproductive sex. In the case of parents, there isn't much information about how sexual behavior connects men and women in the service of provisioning for offspring. One can imagine that feelings of pleasure and well-being would lead to more positive associations in a couple caring for a child or children; this would enable them to better allocate effort as well as forgive one another for errors, asymmetries in effort, or other indiscretions that would, in the case of a less reinforced pair bond, cause one or both parties to abandon the relationship. In the case of pair bonding between parents, a man's pleasure at sexual contact with the mother of his children may prevent him from allocating his effort or resources to other mating opportunities or other children. For a woman, continued sexual pleasure with the father of her children could prevent her from abandoning a relationship that is provisioning and securing her offspring and could act as a signal of assurance of paternity to a mate provisioning offspring and offering protection. Thus, orgasm may act as a safeguard and mechanism for forgiveness of defection. The outcome of having healthy offspring that survive to adulthood is distal but the pleasure in the pair bond facilitates it in the short term. These kinds of factors maintaining parental bonds may not be that important in the modern age, but in many hunter gatherer groups thought to resemble ancestral human societies, children without paternal provisioning have lower survivability (Hurtado & Hill, 1992).

Oxytocin is thought to be important for romantic bonding and is released during close affectionate and sexual behavior and most importantly during orgasm. Oxytocin is positively reinforcing (László et al., 2016), increases the sensitivity of non-human primates to positive reinforcement, and increases the reinforcing properties of seeing another monkey reinforced (Chang, Barter, Ebitz, Watson, & Platt, 2012). Oxytocin may increase the strength of orgasm and is released in amounts based on the quality of orgasm (Behnia et al., 2014) and increases the sensitivity to socially reinforced (e.g. smiling faces) learning (Hurlemann et al., 2010). However, the fact that sexual excitement and satisfaction decreases over the course of a relationship and are greater with novel partners implies that orgasm is not intended to pair bond couples indefinitely but may only improve pair bonding for the duration in which children need intensive care and provisioning (H. E. Fisher, 1989).

The pair bonding hypothesis of orgasm has been supported and contradicted by evidence for and against the association of orgasmic frequency and relationship satisfaction. Coital (but not non-coital) orgasm frequency has been found in some studies to correspond to good dimensions of relationship quality such as intimacy, passion, love, and satisfaction (Costa & Brody, 2007). However, other studies have found that attractiveness but not positive dimensions of relationship quality account for orgasm (orgasm during last coitus) (Shackelford et al., 2000). If one considers attachment and affection to another person as a behavior that can be reinforced with orgasm, we might not expect consistent orgasmic and sexual pleasure. With animals, a behavior that is being successfully and consistently produced need not be reinforced consistently (e.g. a dog sitting), but behavior that is decreasing in frequency may need to be carefully shaped when any subtle cue of that behavior reappears. A waning behavior like affection or attachment may be more effectively strengthened by, for example, rewarding the desired cues with variable reinforcement (e.g. the slot machine example used earlier) (Pryor, 1999; Skinner, 1938). We might expect orgasm would reinforce the attachment of two people after a period of divestment. For example, if orgasm serves to reinforce closeness and positive association between two people, we might expect intense sexual pleasure, orgasm, and intense orgasm to occur after relationship stress (e.g. threat from a rival, absence from a partner) rather than during every sexual episode. This could be a complementary reason why men find cues of other males mating with their mate sexually arousing (Pound, 2002), not just to engage in sperm competition but to strengthen the association between proximity and sexual pleasure to their mate to reignite the pair bond. Thus, we should predict that strong pair bonds should not exhibit regular orgasmic frequency but orgasm may instead track the degree to which strong reinforcement would be adaptive.

Another piece of evidence for orgasm as a pair bond mechanism is that foreplay increases the rate of orgasm in women (Singh, Meyer, Zambarano, & Hurlbert, 1998). This may indicate that foreplay classically conditions women to be secondarily reinforced by increased proximity to their partner as well as their time and attention. This could also indicate that orgasm as a signal is a reinforcement, a consequence that operantly conditions devoting attention and time to the partner (but more on that later). Thus far, the evidence indicates that an attractive and dominant male may increase the rate of orgasm in women. Dominant men may also have the time, experience, and confidence to engage in more extensive foreplay without being interrupted by rivals. It seems that foreplay can be used by dominant men to cultivate the pair bond with an especially attractive partner (as this will entail fewer opportunity costs for copulating with others), but that lower status males may also be able to use the same strategy: time, attention, and knowledge of a partner to increase attachment and reinforce the pair bond.

Let us now briefly consider orgasm and sexual pleasure from the perspective of forming and maintaining bonds between men and women for various benefits other than directly provisioning offspring. One might predict that the pleasure of sex and orgasm would be more associated with relationships that optimally conferred status, resources, or safety as a way of reinforcing association with that social partner and as a way of sexually reinforcing that social partner's association with you. It's difficult to disentangle orgasm in response to ‘good genes’ (e.g. characteristics like attractiveness and masculinity), as these characteristics also are likely to confer high status in social groups and lead to social and resource benefits of association regardless of whether sex causes conception with these men. One example of using sexual pleasure and orgasm to garner social benefits is the case of female baboons forming sexual friendships with males (Smuts, 1985). The female allows sexual access to a male while she is in estrus and that male protects her and her offspring. This isn't a perfect example because the friendships are platonic between estrus periods and maintained by proximity and grooming. However, we could say that the female's sexual pleasure reinforces proximity with a male who protects her and the male's sexual pleasure reinforces attention and care for the female. Among human opposite sex friendships, both men and women want opposite sex friends who are agreeable and dependable, but men tend to prefer opposite sex friends who are attractive, and women tend to prefer opposite sex friends who have resources and physical strength (Lewis, Conroy-Beam, Raja, Dekay, & Buss, 2011). Sexual pleasure and orgasm, but also mere proximity to attractive cues, may maintain such relationships. Furthermore, when one gives sexual pleasure to a social partner, this increases his or her preference for you over other friends when there is competition for his or her status, attention, or other resources.

Orgasm is highly complex and occurs in a variety of sexual contexts. Although sire choice can encompass many aspects of reproduction (e.g. orgasm with a symmetrical male), the so-called ‘sire choice’ hypothesis of orgasm posits that orgasm causes women to ‘preferentially retain’ sperm of higher genetic quality (Sela et al., 2015). However, orgasm, seems overly complex in our species to have evolved to mainly solve this particular problem. To summarize, I tend to agree with Hrdy (1996): ‘why solve a (presumably relatively simple?) cell transport problem through the evolution of a complex psychophysiological phenomenon that requires selection pressures to elaborate and link up various organs’. Orgasm seems well-designed as a mechanism to increase copulation among genetically desirable mates. However, we would also expect that evolution would select against orgasm and sexual pleasure in other contexts that didn't facilitate direct fertilization if they were maladaptive. Orgasm occurs in many different contexts and with people both of the same sex and the opposite sex; this may indicate that some non-reproductive sexual behavior is adaptive.

## Same-sex sexual behavior and orgasm

For many species, there is no motivation for sex or pleasure in sex if there is no chance for reproduction; evolution is capable of eliminating the pleasure of behaviors, including sexual behaviors, if there is no adaptive benefit. This is one reason why same-sex sexual behavior has been seen as a mystery to biology and psychology; how can a behavior that offers no possibility of direct reproductive success be maintained in the population? However many people feel the answer to the following question is obvious: Why might opposite sex partners engage in sexual behavior that has no possibility of reproduction (e.g. the woman is already pregnant or one partner is past fertile age)? It's likely we would say they engaged in the act to increase their intimacy or to give one another pleasure. It also stands to reason that bonds between people of the same sex (homosocial) can be strengthened with sexual behavior. Affiliating with others and engaging in cooperative exchange activates neural reward centers in the brain (Bora, Yucel, & Allen, 2009). These interactions can be even more reinforcing if they include associations of sexual pleasure.

The affiliation hypothesis of homosexual behavior (Fleischman, Fessler, & Cholakians, 2015) proposes that selection used the pleasure of sexual behavior to promote same-sex social bonds. In many societies that are thought to resemble social groups throughout human evolutionary history, social bonding and alliances play a critical role in survival. Some examples include defense in violent conflict (Van Vugt, 2009), surviving food shortfalls (Hill & Hurtado, 2009), care during illness or injury (Sugiyama, 2004), and cooperative child rearing (alloparenting) (Hrdy, 2009). Among humans and non-human primates, social bonds translate into better survival and reproductive success (Silk, Alberts, & Altmann, 2003; Silk et al., 2010). In the toolkit of means of reinforcement, sexual pleasure and orgasm would have been very useful both with members of the same sex, and members of the opposite sex, for cementing and maintaining social bonds. Evolution maintains sexual pleasure for a diversity of sexual behavior among humans and facilitates motivation to engage sexually to different adaptive ends.

## Classical and operant conditioning and orgasm

Classical conditioning, as discussed earlier, is a process by which a neutral stimulus is paired with a primary reinforcer, like sexual pleasure or orgasm, which thereby makes the neutral stimulus in itself reinforcing. When two people engage in sexual behavior and have orgasms they are associating sexual pleasure with the characteristics of the other including proximity, smell, taste, and form; these all become secondary rewards/reinforcers. When two people have repeated erotic contact, they become classically conditioned to perceive one another as secondary reinforcers and can better shape one another toward their own strategic goals.

If one person in a dyad is associated with sexual pleasure, he or she can very easily engage in what is called ‘negative punishment’, withholding positive consequences for a behavior as a means of punishment and an adaptive tactic for extinguishing the behavior. Take, for example, ‘silent treatment’; If an individual values social interaction with a sexual partner, then taking away this reinforcer is a way of simultaneously punishing the behavior and no longer supplying positive reinforcement toward that behavior. Silent treatment will be much more painful if someone's mere presence, eye contact, or voice is strongly secondarily reinforced by sexual pleasure. Furthermore, withdrawal of other stimuli often associated with orgasm like touch, eye contact, close proximity, and smell can be used more subtly (than silent treatment) to shape behavior both consciously and unconsciously.

Orgasm can also be used more directly to operantly condition behavior with or without classical conditioning. Operant conditioning is a process by which a behavior is followed by positive or negative consequences that increase or decrease the frequency of the behavior. An animal will engage in a behavior for a food reward and increase the frequency of that behavior if food rewards continue.

## The evolutionary psychology of dyadic social shaping others’ behavior as an extended phenotype

In ‘The Extended Phenotype’, Dawkins advances the hypothesis that those things that are proximal to an organism, their physiological characteristics, behavior patterns, and individual differences are not the only things that make up their phenotype (Dawkins, 1999). A phenotype also consists of an organism's effects on the environment, including its effects on other organisms. One good example of this is parasitic manipulation. *Toxoplasma gondii* has an extended phenotype that includes rat psychology. This protozoa causes rats to increase their attraction to the smell of cat urine thus facilitating being eaten by cats, Toxoplasma's primary host (Berdoy, Webster, & Macdonald, 2000). In a more sexually relevant example, the ‘Bruce effect’ is a pregnancy disruption that occurs when a female mouse smells an unfamiliar male mouse's pheromones (Dawkins, 1999). In this case, her pregnancy is immediately terminated and she becomes sexually receptive and fertile much more quickly than if she had carried her litter to term. Dawkins argues that one does not have to view the abortion and early receptivity of the female mouse as only an adaptation on her part but can also view this as an adaptation on the part of the male mouse. Manipulation of anything external, including the manipulation of other organisms and conspecifics, is under just as intense selection pressure as those phenotypic characteristics that enable the organism to adapt to its immediate environment. Some of the physical and social environment represents an organism's phenotype, and humans are an example par excellence of a species that shapes its social environment in myriad ways (e.g. niche construction). Orgasm and sexual arousal can be used to extend one's phenotype into the minds of others in order to manipulate them to one's own strategic goals.

Between any two people, interests will sometimes align and sometimes diverge. The adaptive interests of individuals can be furthered with a variety of strategies; other individuals can engage in *strategic interference* or *strategic facilitation* (Buss, 1989). One of the most fraught kinds of relationships where there is the most at stake is between romantic partners whose interests can diverge in important ways. For example, in the case of jealousy, negative emotions are evoked to punish the romantic partner. In the case of men, jealousy is more often evoked in the case of sexual infidelity as it indicates that men may have heavily invested in offspring that are not genetically related to them (Buss, Larsen, Westen, & Semmelroth, 1992). In this case, a man may punish a woman in a variety of ways for having sex outside of the pair bond because it interferes with his adaptive strategy of investing in offspring that are genetically related to him. Women are slightly more likely to experience emotional jealousy indicating the possible divestment of resources away from her or her offspring. She may punish the man for divesting resources to another woman as she wants to dissuade him from interfering with her strategy of obtaining resources and security from her romantic partner. When individuals engage in sexual behavior, they increase the leverage they have to both reinforce and punish behaviors to promote strategic facilitation and interfere with strategic interference.

A variety of tactics can be used to reinforce and punish others, especially if you are a secondary reinforcer to someone because of classical sexual conditioning. While this hasn't been explored directly in the literature before, there is one paper that details possible ‘manipulation tactics’ that can be used between romantic partners (Buss, 1989): charm, silent treatment, coercion, reasoning, regression, and debasement. Each of these can be viewed from a social shaping perspective and most could be augmented with the association of sexual pleasure. The ‘charm’ tactic includes acts of love and affection, compliments, gifts, and promising a reciprocal favor in exchange for a behavior in the mate, other means of operantly and classically conditioning a partner than sexual behavior. We already discussed silent treatment above as an example of negative punishment.

Here, I want to stress that positive association between a behavior and a reinforcing outcome like sexual pleasure is unlikely to be overt and, in fact, may work better for strategic goals if the manipulation is covert or unconscious. Self-deception is likely at play here; people are rarely conscious of the ways they manipulate others, and consciously verbalizing how you are manipulating someone else through reinforcement, would likely be viewed as unethical or sociopathic. The tactics of manipulation people use both sexually and non-sexually very likely exceed those they can verbalize. It doesn't seem that people are either often consciously aware of how they have sex to reinforce behavior or that they are willing to admit this in questionnaires. For example, individuals report engaging in sex for attraction or pleasure or to say ‘I've missed you’ very frequently, but very infrequently report that they have sex to manipulate another person into a given behavior (Meston & Buss, 2007). Perhaps, there has been selection for ignorance of one's own manipulation tactics so that they have greater effectiveness.

This is an area that could be important for further study. For example, having sex because you've missed another person or to make up after a fight may be a way to orgasmically reinforce their proximity, the dissolution of their anger, or their investment in the relationship (whether this takes the form of resources or security). However, it is not only the pleasure of orgasm that reinforces behaviors; seeing another person have an orgasm can be in itself reinforcing and a signal of sensitivity to reinforcement and punishment.

## Orgasm as a signal of sensitivity to shaping

Given what has been discussed so far in this paper, it would be surprising if cues of intense sexual pleasure and orgasm in another person were not themselves primary reinforcers. Perceiving that you have given someone else intense sexual pleasure can be a signal of trainability. Seeing intense sexual pleasure in another person is an indication of the strength of the extension of your phenotype into their minds, like altering a landscape to bear fruit. This may be yet another reason that orgasm is less frequent in women than it is in men. If men have, over evolutionary history, experienced paternity uncertainty, they would not only be sensitive to women's fidelity but also sensitive to the extent that their partner was specifically sensitive to receiving pleasure from them as a sexual partner and thus more reinforced by their company. If one had a precious resource in a lockbox, one would feel more secure if that lockbox had fewer combinations capable of opening it. Similarly, if one's sexual partner is intensely reinforced by one's sexual stimulation, this indicates that they are, potentially, uniquely reinforced to the exclusion of others and therefore one's investment is secure. This also indicates that they needn't try to maintain as intense a monopoly on sexual access through ‘mate guarding’. Indeed, men's relationship satisfaction is positively predicted by their partner's orgasmic behavioral intensity (Ellsworth & Bailey, 2013). However, this could explain why giving sexual pleasure with any partner may hold intrinsic importance but especially in cases where strategic facilitation has the most adaptive value, as between reproductive partners. Of all the relationships humans have with one another, romantic relationships often result in the most shared genetic fate. Because the behavior of one's mate may have such a strong impact on one's reproductive success, tactics of shaping behavior should be heavily employed in the context of romantic relationships from the initial stages of courtship to managing parental duties and deterring the divergence of resources. Women's orgasmic behavioral intensity is positively related to their perception of partner investment (Ellsworth & Bailey, 2013). This also helps explain why orgasm is sometimes faked (Brewer & Hendrie, 2011), as it is likely both a reinforcing signal and a signal of special reinforcement sensitivity. Women (and more rarely men) may want to deceive their partners to the extent to which they are attached, trainable, and reinforcable. Relatedly, even though women are more likely to experience emotional jealousy, we should expect that the intensity of sexual pleasure and orgasm of a woman's mate with another partner should elicit acute jealousy because of its likelihood of eliciting greater attachment through classical conditioning.

## Conclusion

This paper has been an overview of a potentially overlooked framework for elucidating sexual behavior. One reason for this may be that self-deception is involved in shaping others. This framework helps explain why social bonds and influence can be amplified with the addition of sexual pleasure and, especially, orgasm. It also explains why we, as humans, have so much attention and memory for the kinds of pleasure our partners find most reinforcing and intense. Many of these ideas have yet to be explored through scientific investigation. Do people find ‘silent treatment’ or distance most painful from those that give them the most sexual pleasure? Does one find the signal of orgasm most reinforcing from those that are the most strategically important to one's adaptive goals? How does sexual pleasure reinforce bonds between people of the same sex in different contexts? Explicitly using the psychology of learning and social shaping can expand an evolutionary account of sexual behavior and the myriad reasons it is adaptive both for conception, stronger social bonds, and strategic facilitation.
